# Evaluation of alginate/poly(vinyl alcohol)/BaSO_4_ hydrogels for nucleus pulposus regeneration

**DOI:** 10.1039/d5ra04291g

**Published:** 2025-09-22

**Authors:** Amaliya Rasyida, Dindra Fai'mundiarti Purnamabroto, Agung Purniawan, Indra Carllistya Pramadio, Femiana Gapsari, Adelina Salsabila Ednanda

**Affiliations:** a Department of Materials and Metallurgical Engineering, Faculty of Industrial Technology and Systems Engineering, Institut Teknologi Sepuluh Nopember Surabaya 60111 Indonesia amaliya@its.ac.id; b University of Brawijaya Malang Indonesia

## Abstract

Intervertebral disc degeneration is a major contributor to lower back pain worldwide, underscoring the urgent need for effective, minimally invasive regenerative therapies. One of the critical challenges in nucleus pulposus (NP) replacement lies in developing injectable hydrogels with optimal gelation behavior, radiopacity, mechanical properties, and biocompatibility. This study investigates the influence of varying concentrations of Na_2_HPO_4_ (0.3%, 0.4%, 0.5%) and BaSO_4_ (1%, 1.5%) in ALG/PVA hydrogels through a series of gelation time measurements, radiopacity analysis, mechanical testing, and *in vitro* biocompatibility assays. The optimal formulation, containing 1.5 wt% BaSO_4_ and 0.4 wt% Na_2_HPO_4_, achieved a gelation time of 12.5 ± 0.5 minutes, radiopacity of 71–74%, elastic modulus of 0.055 ± 0.015 MPa, and cell viability above 90%, fulfilling key criteria for NP scaffold performance. Although compressive strength remained below physiological requirements, the formulation demonstrated excellent injectability, structural integrity, and biological response. This study offers a novel strategy by combining retarding and radiopaque agents in a single hydrogel system, contributing to the advancement of injectable biomaterials for intervertebral disc regeneration.

## Abstract 2

Intervertebral disc degeneration is a major contributor to lower back pain worldwide, underscoring the urgent need for effective, minimally invasive regenerative therapies.

## Graphical abstract

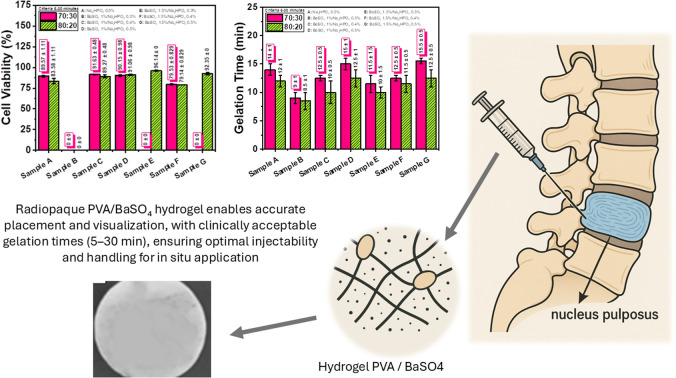

## Introduction

Spinal disorders are a growing public health concern, affecting over 632 million people globally and exerting a substantial socio-economic burden due to their chronic nature and high treatment costs. Among these conditions, lower back pain is the most prevalent, commonly caused by degenerative disc disease (DDD) or intervertebral disc degeneration (IVD). The intervertebral disc (IVD) is composed of three main structural components: the nucleus pulposus (NP) at the center, which provides hydration and mechanical cushioning; the surrounding annulus fibrosus (AF), which provides tensile strength; and the cartilage endplate (CEP), which facilitates nutrient transport. The NP plays a critical role in maintaining spinal flexibility and load distribution. Degeneration of this gelatinous tissue can lead to reduced disc height, structural instability, nerve compression, chronic pain, and limited mobility. Lower back pain emerges as a complex and multifactorial condition, driven by anatomical degeneration, age-related changes, and lifestyle factors, necessitating effective regenerative strategies^1–4^

Historically, the concept of NP replacement was introduced in the 1960s by Nachemson,^5^ who injected self-curing silicone into cadaveric discs. This approach evolved in the early 1990s when Bao and Higham^6^ developed injectable hydrogels as biomimetic substitutes for NP. These materials have since gained traction due to their ability to conform to irregular defects, reduce implant migration, and minimize surgical invasiveness. The biological and mechanical criteria for successful NP replacement include biocompatibility, biodegradability, sufficient mechanical strength, controlled gelation time, and radiopacity. Injectable hydrogels now play a pivotal role in tissue engineering, acting as scaffolds that restore disc structure and function^7–9^

Nevertheless, several challenges hinder the clinical efficacy of injectable hydrogels for NP regeneration. Chief among these are their insufficient compressive strength (ideally ∼1.0 MPa), unpredictable gelation kinetics, and a high risk of post-injection leakage. These issues can impair the integration and performance of hydrogels *in vivo*, particularly under the dynamic and pressurized conditions of the intervertebral disc. Hence, advanced hydrogels must exhibit finely tuned mechanical, biological, and radiological properties. A strategic approach involves incorporating retarding agents, such as Na_2_HPO_4_, to delay premature gelation, and radiopaque agents, such as BaSO_4_, to improve visualization and placement accuracy during clinical procedures.^10–14^

Among potential biomaterials, alginate is widely recognized for its biocompatibility, biodegradability, and structural similarity to natural extracellular matrix (ECM), making it an ideal candidate for NP scaffold design. It supports cell proliferation and ECM synthesis under physiological conditions. When combined with poly(vinyl alcohol) (PVA), a co-polymer known for its mechanical robustness, partial biodegradability, and good hydrogel-forming capacity, the resulting composite offers enhanced mechanical stability while retaining biological functionality. In this context, Na_2_HPO_4_ serves as a retarding agent to regulate ionic crosslinking between alginate and calcium ions, thus optimizing gelation time. Concurrently, BaSO_4_ functions as a radiopaque additive, enabling radiological imaging during and after injection, thereby facilitating real-time monitoring and post-operative assessment.^15–17^

This study aims to systematically investigate the effects of varying Na_2_HPO_4_ (0.3%, 0.4%, and 0.5 wt%) and BaSO_4_ (1% and 1.5 wt%) concentrations in ALG/PVA hydrogels, with specific focus on gelation time, radiopacity, porosity, swelling behavior, compressive strength, and biocompatibility. The novelty of this research lies in the dual incorporation of both retarding and radiopaque agents—a combination that has not been extensively explored in previous studies, which often examined these additives independently. The findings of this study are expected to contribute significantly to the development of optimized injectable hydrogel systems that meet the clinical requirements for NP regeneration, potentially improving therapeutic outcomes in patients with IVD degeneration and expanding the applications of these biomaterials in spinal tissue engineering.^18–30^

## Experimental procedure

### Materials and methods

#### Hydrogel synthesis

The ALG/PVA/BaSO_4_ composite hydrogel was synthesized by dissolving poly(vinyl alcohol) (PVA) at concentrations of 20 wt% and 30 wt% in 40 mL of distilled water. The solution was stirred using a magnetic stirrer at 120 °C and 1000 rpm for 1 hour until complete dissolution was achieved. Once a homogeneous solution was obtained, BaSO_4_ powder was added at 1 wt% and 1.5 wt% concentrations, followed by stirring for 10 minutes. Sodium alginate (SA) was then added at 80 wt% for the 20 wt% PVA group and 70 wt% for the 30 wt% PVA group, and the mixture was stirred at 250 rpm for 1 hour. Di-sodium hydrogen phosphate (Na_2_HPO_4_), pre-dissolved in 5 mL of distilled water, was added at concentrations of 0.3 wt%, 0.4 wt%, and 0.5 wt%, and stirred at 250 rpm for 45 minutes. Additionally, calcium sulfate (CaSO_4_) powder (1 wt%) was dissolved in 5 mL of distilled water and stirred at 250 rpm for 45 minutes to form a CaSO_4_ suspension.

#### Materials

The following reagents were used: sodium alginate (medium viscosity, 80–120 kDa), PVA (*M*_w_ = 60 000 g mol^−1^), calcium sulfate (CaSO_4_, *M*_w_ = 136.14 g mol^−1^), Na_2_HPO_4_ (*M*_w_ = 177.99 g mol^−1^), and BaSO_4_ (*M*_w_ = 233.40 g mol^−1^). Sodium alginate and PVA were sourced from Sigma-Aldrich (USA) and Merck (Germany), respectively; CaSO_4_ from MaxLab (Indonesia); and both Na_2_HPO_4_ and BaSO_4_ from SAP Chemicals (Indonesia).

#### Gelation time measurement

Gelation time was evaluated to determine the duration required for the composite hydrogel to transition into a non-flowable gel state. Samples were prepared in cylindrical molds with dimensions approximating 10 mm in diameter and 5 mm in height, mimicking the size of a native nucleus pulposus. The surface gelation time (when the top layer no longer flows upon tilting) and full-body gelation time (when the entire sample solidifies uniformly) were recorded using a stopwatch under ambient room temperature (∼25 °C) through visual observation. This assessment was essential for validating the applicability of the hydrogel for injectable systems. Previous studies report that alginate-based hydrogels exhibit gelation times between 5–30 minutes, suitable for biomedical applications such as tissue engineering and wound healing.^19,31–33^

#### Fourier transform infrared spectroscopy (FTIR)

To characterize the chemical structure of the hydrogel, FTIR spectra were acquired using a Thermo Scientific Nicolet IS10 instrument over a wavenumber range of 400–4000 cm^−1^, with a resolution of 4 cm^−1^ and 32 scans per sample. The purpose of the analysis was to identify characteristic functional groups such as hydroxyl (O–H), carbonyl (C=O), and ether (C–O–C) bonds associated with PVA and alginate components.

#### Scanning electron microscopy (SEM) and energy-dispersive X-ray spectroscopy (EDS)

Freeze-dried hydrogel specimens were prepared in cube-shaped samples measuring 1 cm × 1 cm × 1 cm, sputter-coated with platinum for 90 seconds at 20 mA to produce a ∼10 nm coating. The specimens were then analyzed using a FEI INSPECT S50 operating at 15 kV, equipped with an EDS system. SEM imaging was performed to observe surface morphology and porosity, while EDS analysis was used to detect the spatial distribution of key elements, particularly Ba (from BaSO_4_), Na (from Na_2_HPO_4_), and S (from CaSO_4_), to confirm the homogeneity and integration of inorganic fillers in the hydrogel matrix.

#### Radiopacity testing

X-ray imaging was performed using a Shimadzu X-200 radiography unit at 60 kV and 5 mA on disc-shaped hydrogel samples with a diameter of 10 mm and a thickness of 2.5 mm. Radiographic images were analyzed using Adobe Photoshop (Histogram Tool) to evaluate radiopacity by measuring the mean grayscale brightness values on a 0–255 scale, where higher values indicated greater radiopacity. For consistency, each sample was measured in triplicate using a fixed Region of Interest (ROI) to minimize background variation.

#### Compressive strength testing

The compressive strength of the hydrogel samples was evaluated using a Universal Testing Machine (Hung TA Model HT-2402 series 4035) with a crosshead speed of 1 mm min^−1^. The specimens were cylindrical in shape, with a diameter of 10 mm and a height of 20 mm. Testing was performed at room temperature, and values were averaged over three replicates to ensure reliability.

#### MTT assay for biocompatibility evaluation

The *in vitro* biocompatibility of the hydrogel was assessed using the MTT assay. Osteoblast-like 7F2 mouse cells (ATCC® CRL-12557™) were cultured in 96-well plates at a density of 1 × 10^4^ cells per well for 24 hours, followed by treatment with hydrogel samples at a concentration of 10 mg per mL per well. After removal of the culture medium, cells were incubated with MTT solution (0.5 mg mL^−1^ in DMEM) for 4 hours at 37 °C. The resulting formazan crystals were dissolved in dimethyl sulfoxide (DMSO), and absorbance was measured at 570 nm using a Bio-Rad iMark™ microplate reader. Cell viability was calculated by comparing absorbance values of treated samples with those of the untreated control group, where higher absorbance indicated greater viability.

#### Swelling and biodegradation tests

To assess the hydrogel's swelling behavior and biodegradability, samples were first freeze-dried using standard lyophilization techniques at −50 °C for 24 hours. The dry weights of the specimens were recorded, and the samples were immersed in phosphate-buffered saline (PBS, pH 7.4) at 37 °C to simulate physiological conditions.^34^ At predetermined time intervals (1, 3, and 5 hours), the hydrogels were removed, gently blotted using filter paper to remove excess surface water, and weighed. The swelling ratio (*Q*) was calculated as:

1 *Q* = (*W*_S_ − *W*_0_)/*W*_0_

where *W*_S_ is the weight of the swollen hydrogel at time *t*, and *W*_0_ is the initial weight of the dry hydrogel.^35^

For biodegradation analysis, swollen samples were incubated in PBS at 37 °C over the same time intervals (1, 3, and 5 hours). After each time point, samples were dried under vacuum and reweighed to determine the percentage of weight loss, indicating hydrogel degradation.^36^ These evaluations provide insights into the hydrogel's environmental responsiveness, drug delivery potential, and biodegradability under physiological conditions.^37^

## Results and discussion

### Gelation time analysis

**Fig. 1:**
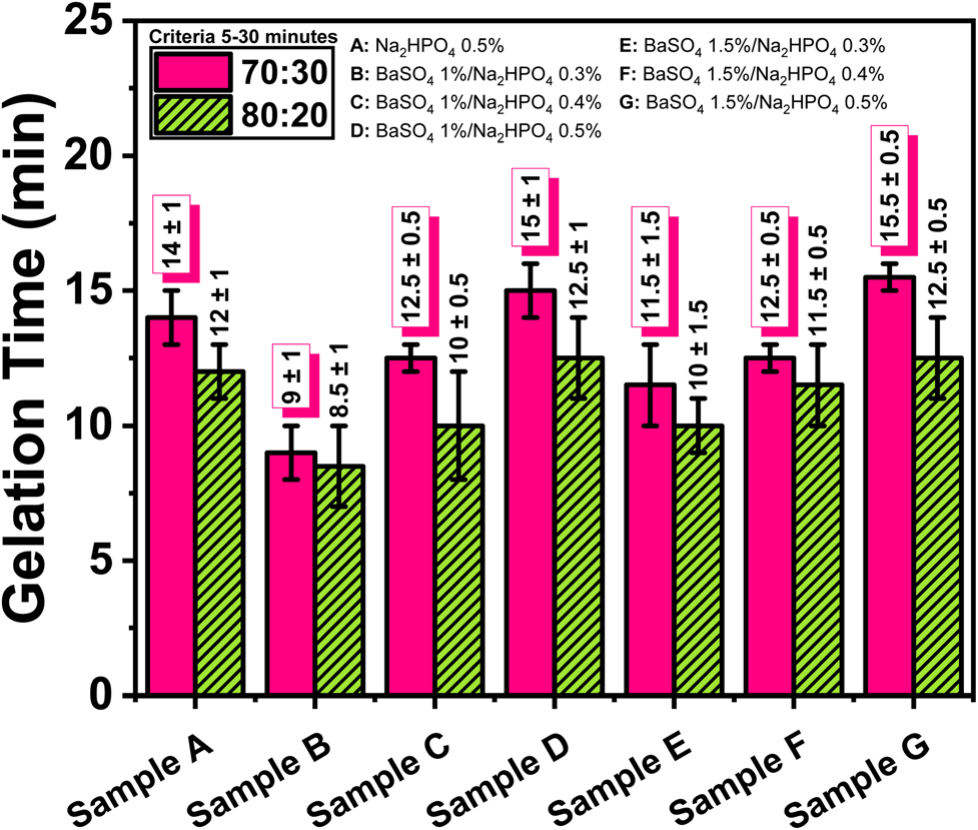
Effect of Na_2_HPO_4_ and BaSO_4_ addition on the gelation time of ALG/PVA/BaSO_4_ hydrogel composites.

Gelation time measurements were performed after pouring the synthesized hydrogel into nucleus pulposus molds. The data presented in Fig. 1 indicate that the sample with 1 wt% BaSO_4_ and 0.3 wt% Na_2_HPO_4_ achieved the shortest gelation time, with the 70 : 30 ALG/PVA ratio yielding 9.0 ± 1.0 minutes and the 80 : 20 ratio showing 8.5 ± 1.5 minutes. Conversely, the longest gelation times were observed in samples with 1.5 wt% BaSO_4_ and 0.5 wt% Na_2_HPO_4_. This trend demonstrates that gelation time increases with higher Na_2_HPO_4_ concentrations, likely due to the phosphate group's ability to compete with calcium ions, thereby retarding the ionic crosslinking between alginate and CaSO_4_ (Table 1).^20^

**Table 1:** The effect of addition Na_2_HPO_4_ and BaSO_4_ in gelation time of hydrogel composite ALG/PVA/BaSO_4_

Sample	Gelation time (minutes)	
	Criteria 5–30 minutes^37^	
	70 : 30	80 : 20
Na_2_HPO_4_ 0.5%	14.0 ± 1.0	12.0 ± 1.0
BaSO_4_ 1%/Na_2_HPO_4_ 0.3%	09.0 ± 1.0	08.5 ± 1.5
BaSO_4_ 1%/Na_2_HPO_4_ 0.4%	12.5 ± 0.5	10.0 ± 2.0
BaSO_4_ 1%/Na_2_HPO_4_ 0.5%	15.0 ± 1.0	12.5 ± 1.5
BaSO_4_ 1.5%/Na_2_HPO_4_ 0.3%	11.5 ± 1.5	10.0 ± 1.0
BaSO_4_ 1.5%/Na_2_HPO_4_ 0.4%	12.5 ± 0.5	11.5 ± 1.5
BaSO_4_ 1.5%/Na_2_HPO_4_ 0.5%	15.5 ± 0.5	12.5 ± 1.5
Na_2_HPO_4_ 0.5%	14.0 ± 1.0	12.0 ± 1.0
BaSO_4_ 1%/Na_2_HPO_4_ 0.3%	09.0 ± 1.0	08.5 ± 1.5
BaSO_4_ 1%/Na_2_HPO_4_ 0.4%	12.5 ± 0.5	10.0 ± 2.0
BaSO_4_ 1%/Na_2_HPO_4_ 0.5%	15.0 ± 1.0	12.5 ± 1.5
BaSO_4_ 1.5%/Na_2_HPO_4_ 0.3%	11.5 ± 1.5	10.0 ± 1.0
BaSO_4_ 1.5%/Na_2_HPO_4_ 0.4%	12.5 ± 0.5	11.5 ± 1.5
BaSO_4_ 1.5%/Na_2_HPO_4_ 0.5%	15.5 ± 0.5	12.5 ± 1.5

Interestingly, the expectation that higher Ba^2+^ concentrations from BaSO_4_ would accelerate gelation was not met, likely due to a synergistic interaction with Na_2_HPO_4_. While Ba^2+^ ions can act as secondary crosslinkers, the presence of Na_2_HPO_4_ is believed to disrupt Ca^2+^-mediated gelation and reduce Ca^2+^ ion accessibility, possibly due to increased hydrogel matrix densification. This dual mechanism results in prolonged gelation time, aligning with prior observations on BaSO_4_'s role in alginate-based hydrogels.^20,38^ Nonetheless, all hydrogel formulations exhibited gelation times within the clinically acceptable range of 5–30 minutes, which is crucial for maintaining injectability and handling during *in situ* application.^39^

### FTIR analysis

**Fig. 2:**
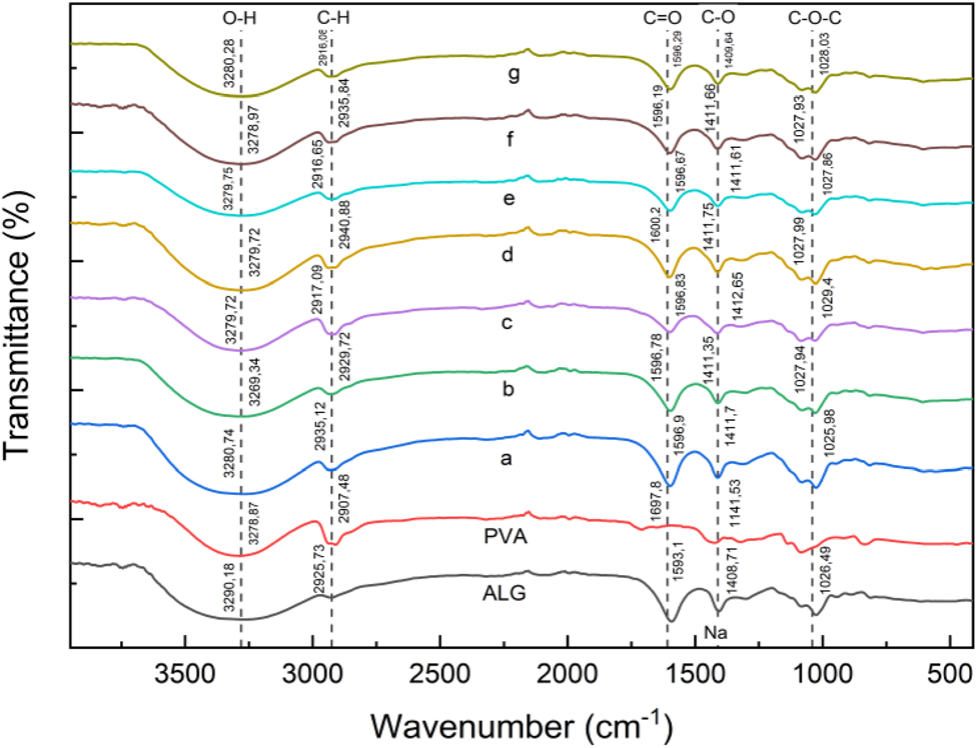
FTIR spectra of ALG/PVA/BaSO_4_ (70 : 30) hydrogels with varying Na_2_HPO_4_ and BaSO_4_ content: (a) Na_2_HPO_4_, 0.5%; (b) BaSO_4_ 1%, Na_2_HPO_4_ 0.3%; (c) BaSO_4_ 1%, Na_2_HPO_4_ 0.4%; (d) BaSO_4_ 1%, Na_2_HPO_4_ 0.5%; (e) BaSO_4_ 1.5%, Na_2_HPO_4_ 0.3%; (f) BaSO_4_ 1.5%, Na_2_HPO_4_ 0.4%; (g) BaSO_4_ 1.5%, Na_2_HPO_4_ 0.5%.

**Fig. 3:**
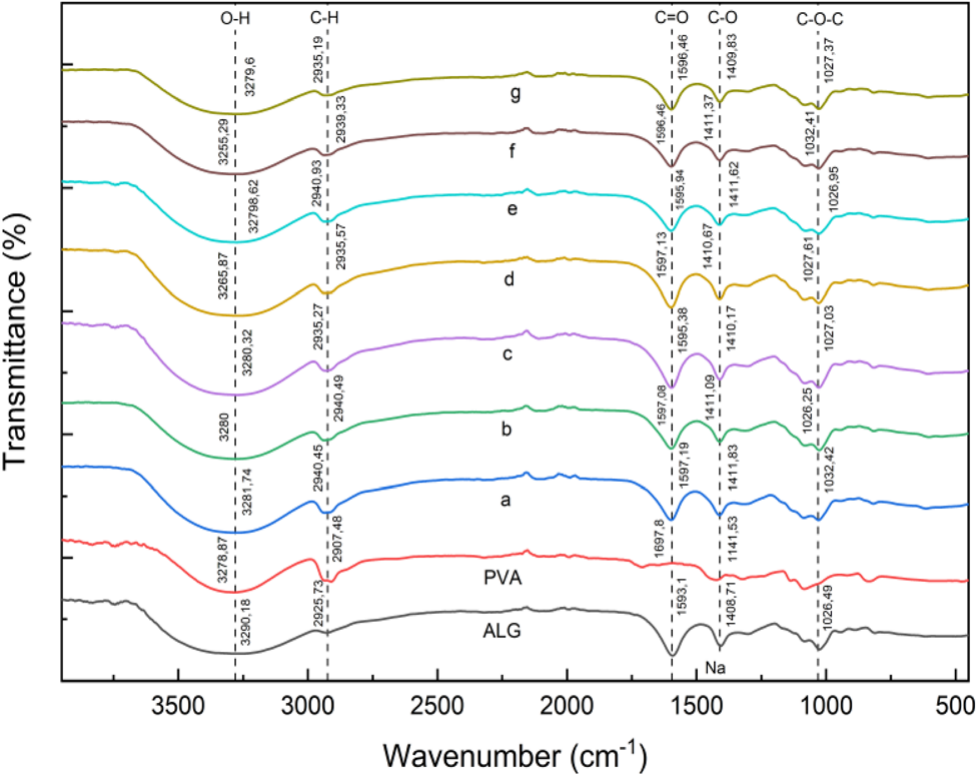
FTIR spectra of ALG/PVA/BaSO_4_ (80 : 20) hydrogels with varying Na_2_HPO_4_ and BaSO_4_ content: (a) Na_2_HPO_4_, 0.5%; (b) BaSO_4_ 1%, Na_2_HPO_4_ 0.3%; (c) BaSO_4_ 1%, Na_2_HPO_4_ 0.4%; (d) BaSO_4_ 1%, Na_2_HPO_4_ 0.5%; (e) BaSO_4_ 1.5%, Na_2_HPO_4_ 0.3%; (f) BaSO_4_ 1.5%, Na_2_HPO_4_ 0.4%; (g) BaSO_4_ 1.5%, Na_2_HPO_4_ 0.5%.

FTIR analysis of the 70 : 30 and 80 : 20 ALG/PVA/BaSO_4_ hydrogels revealed characteristic peaks associated with PVA and alginate functional groups (Fig. 2 and 3). For PVA peaks were identified at 2907 cm^−1^ (O–H), 1697 cm^−1^ (C=O), and 1141 cm^−1^ (C–O). Alginate displayed absorption at 3290 cm^−1^, 2925 cm^−1^, 1593 cm^−1^, 1408 cm^−1^, and 1026 cm^−1^ consistent with O–H, C–H, C=O, C–O–Na, and C–O–C groups respectively. The shift observed in the hydroxyl band suggests intermolecular hydrogen bonding between the alginate and PVA chains, indicating successful polymer interaction during hydrogel formation.^40^

### Morphological analysis (SEM and EDS)

**Fig. 4:**
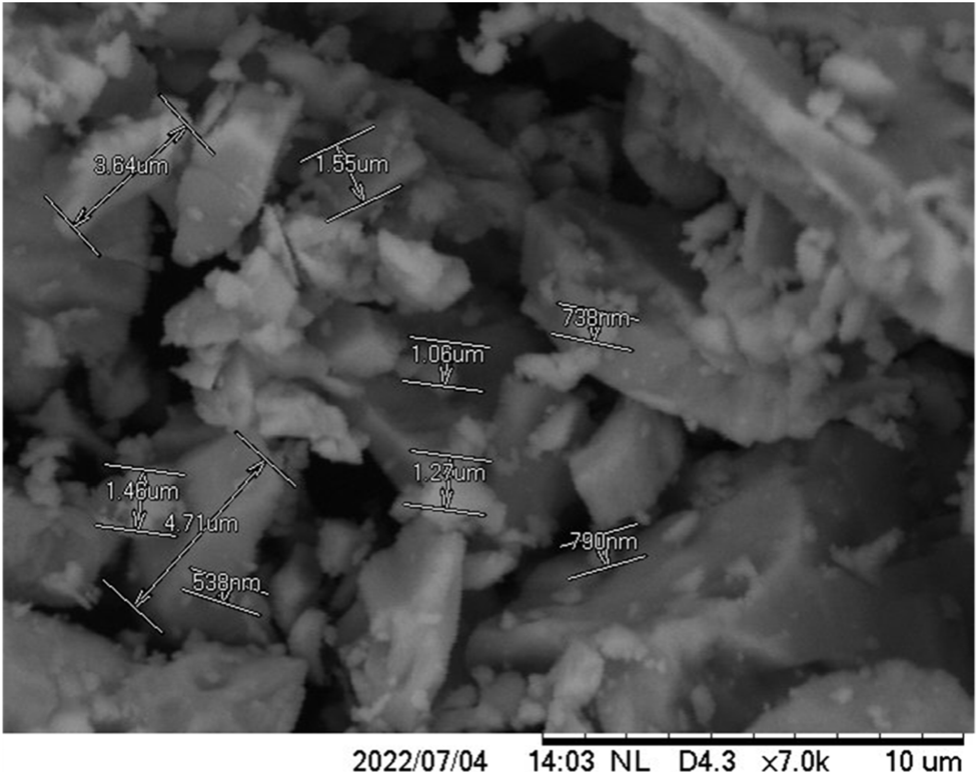
SEM image of BaSO_4_ particles.

**Fig. 5:**
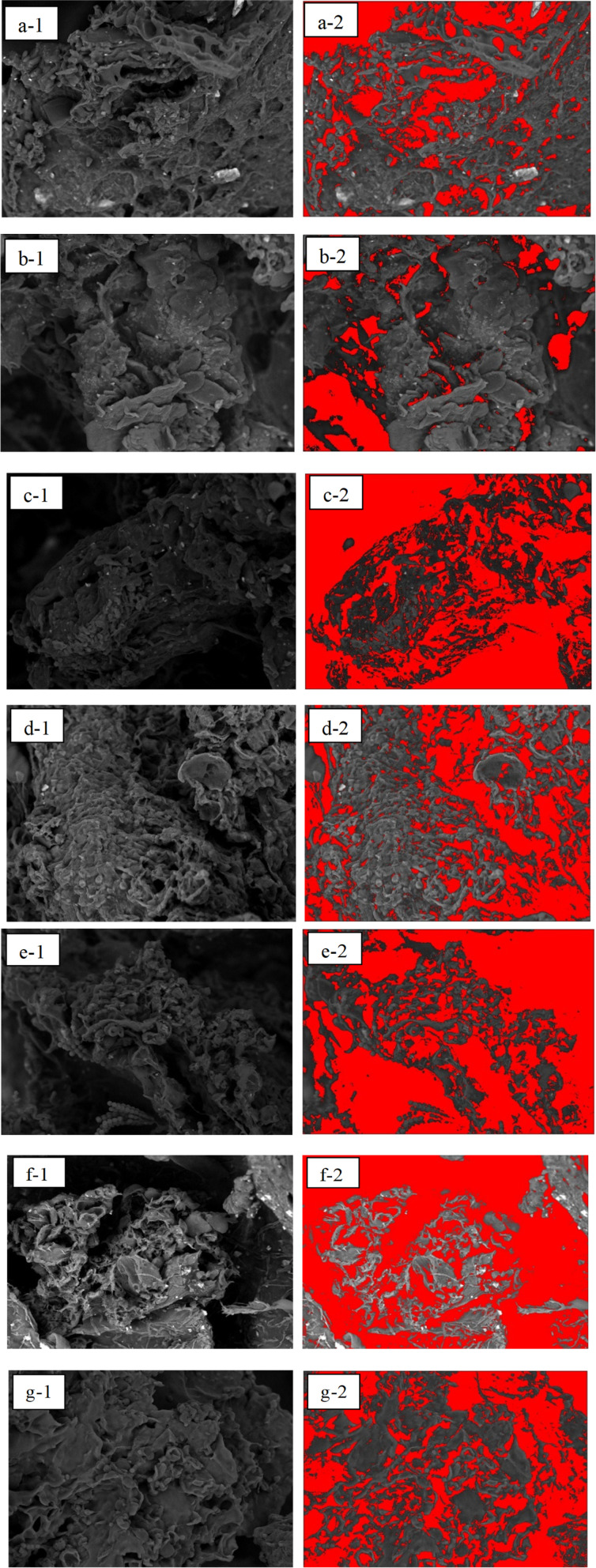
(1) SEM test results for the ALG/PVA/BaSO_4_ hydrogel variation 70 : 30 at 1.000× magnification; (a) BaSO_4_ 1%/Na_2_HPO_4_ 0.3%. (b) BaSO_4_ 1%/Na_2_HPO_4_ 0.4%. (c) BaSO_4_ 1%/Na_2_HPO_4_ 0.5%. (d) BaSO_4_ 1.5%/Na_2_HPO_4_ 0.3%. (e) BaSO_4_ 1.5%/Na_2_HPO_4_ 0.4%. (f) BaSO_4_ 1.5%/Na_2_HPO_4_ 0.5%. (g) Na_2_HPO_4_ 0.5%; (2) the results of pore identification using ImageJ software.

**Fig. 6:**
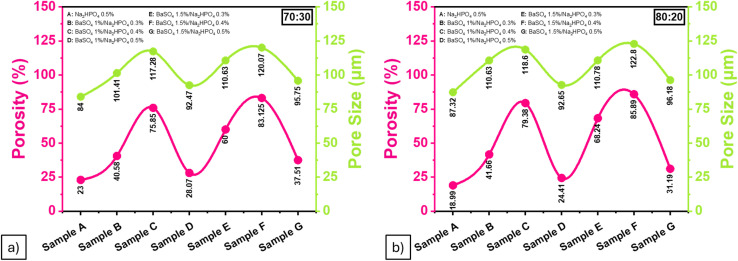
Percent porosity and pore size of ALG/PVA/BaSO_4_ hydrogel composites from (a) 70 : 30, (b) 80 : 20 ratio.

SEM analysis of BaSO_4_ (Fig. 4) showed particle sizes ranging from 538 nm to 4.71 μm with irregular crystalline morphology, which aligns with previously reported characteristics. Before analyzing the composite hydrogels, BaSO_4_ morphology was examined to understand its integration behavior within the matrix. SEM imaging of the 70 : 30 ALG/PVA/BaSO_4_ hydrogel at 1000× magnification (Fig. 5) revealed heterogeneous pore structures. The porosity was quantified using ImageJ software (Fig. 6, Tables 2 and 3). The highest porosity (85.89%) was observed in the sample containing 1.5 wt% BaSO_4_ and 0.4 wt% Na_2_HPO_4_, closely approaching the reported scaffold suitability threshold of 81.28 ± 4.10%.^41^

**Table 2:** Percent porosity of ALG/PVA/BaSO_4_ hydrogel composites

Sample	% porosity	
	81.28 ± 4.10%	
	70 : 30	80 : 20
Na_2_HPO_4_ 0.5%	23	18.99
BaSO_4_ 1%/Na_2_HPO_4_ 0.3%	40.58	41.66
BaSO_4_ 1%/Na_2_HPO_4_ 0.4%	75.85	79.38
BaSO_4_ 1%/Na_2_HPO_4_ (0.5%)	28.07	24.41
BaSO_4_ 1.5%/Na_2_HPO_4_ 0.3%	60	68.24
BaSO_4_ 1.5%/Na_2_HPO_4_ 0.4%	83.125	85.89
BaSO_4_ 1.5%/Na_2_HPO_4_ 0.5%	37.51	31.19

**Table 3:** Pore size of the ALG/PVA/BaSO_4_ hydrogel composites

Sample	Pore size (μm)	
	100–400 μm	
	70 : 30	80 : 20
Na_2_HPO_4_ 0.5%	84	87.32
BaSO_4_ 1%/Na_2_HPO_4_ 0.3%	101.41	110.63
BaSO_4_ 1%/Na_2_HPO_4_ 0.4%	117.28	118.6
BaSO_4_ 1%/Na_2_HPO_4_ (0.5%)	92.47	92.65
BaSO_4_ 1.5%/Na_2_HPO_4_ 0.3%	110.63	110.78
BaSO_4_ 1.5%/Na_2_HPO_4_ 0.4%	120.07	122.8
BaSO_4_ 1.5%/Na_2_HPO_4_ 0.5%	95.75	96.18

Pore sizes between 100–400 μm are optimal for cell attachment and proliferation. Most hydrogel variants met this criterion. However, two specific formulations—those without BaSO_4_ and those containing 1.5 wt% BaSO_4_ with 0.5 wt% Na_2_HPO_4_—exhibited smaller pore sizes that fell below this optimal range (Table 2).

**Fig. 7:**
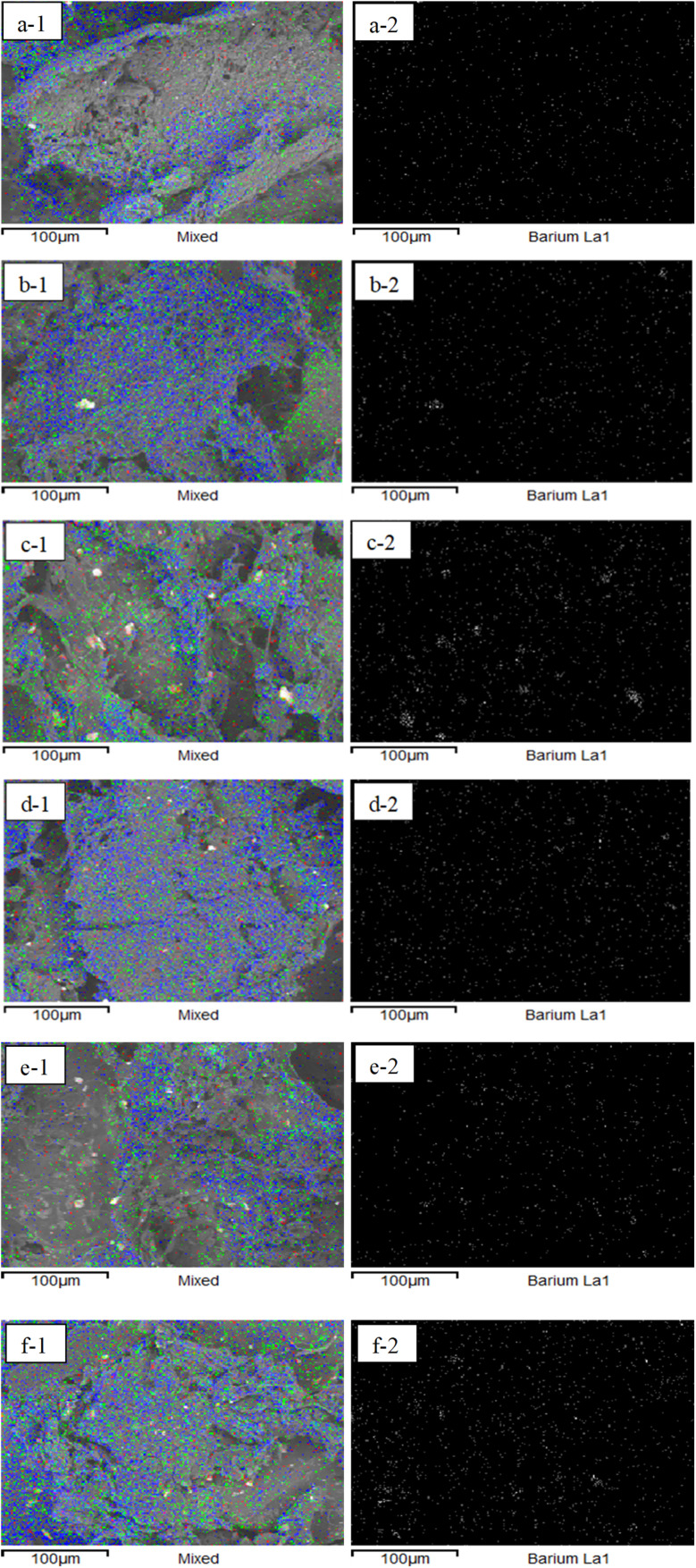
(1) Mapping of elements on ALG/PVA/BaSO_4_ hydrogel variation 70 : 30 [blue is barium, green is sodium, and red is sulfur]; (a) BaSO_4_ 1%/Na_2_HPO_4_ 0.3%, (b) BaSO_4_ 1%/Na_2_HPO_4_ 0.4%, (c) BaSO_4_ 1%/Na_2_HPO_4_ 0.5%, (d) BaSO_4_ 1.5%/Na_2_HPO_4_ 0.3%, (e) BaSO_4_ 1.5%/Na_2_HPO_4_ 0.4%, (f) BaSO_4_ 1.5%/Na_2_HPO_4_ 0.5%; (2) mapping the distribution of barium ALG/PVA/BaSO_4_ hydrogel.

EDS mapping (Fig. 7) confirmed the spatial distributions of Ba, Na, and S, represented by blue, green, and red signals, respectively. However, the observed agglomeration of BaSO_4_ particles reduced their dispersion efficiency within the matrix, which may hinder uniform mechanical reinforcement and cell interaction. This suggests that further interfacial modification could improve matrix-particle bonding and nucleation uniformity.^42^

### Radiopacity evaluation

**Fig. 8:**
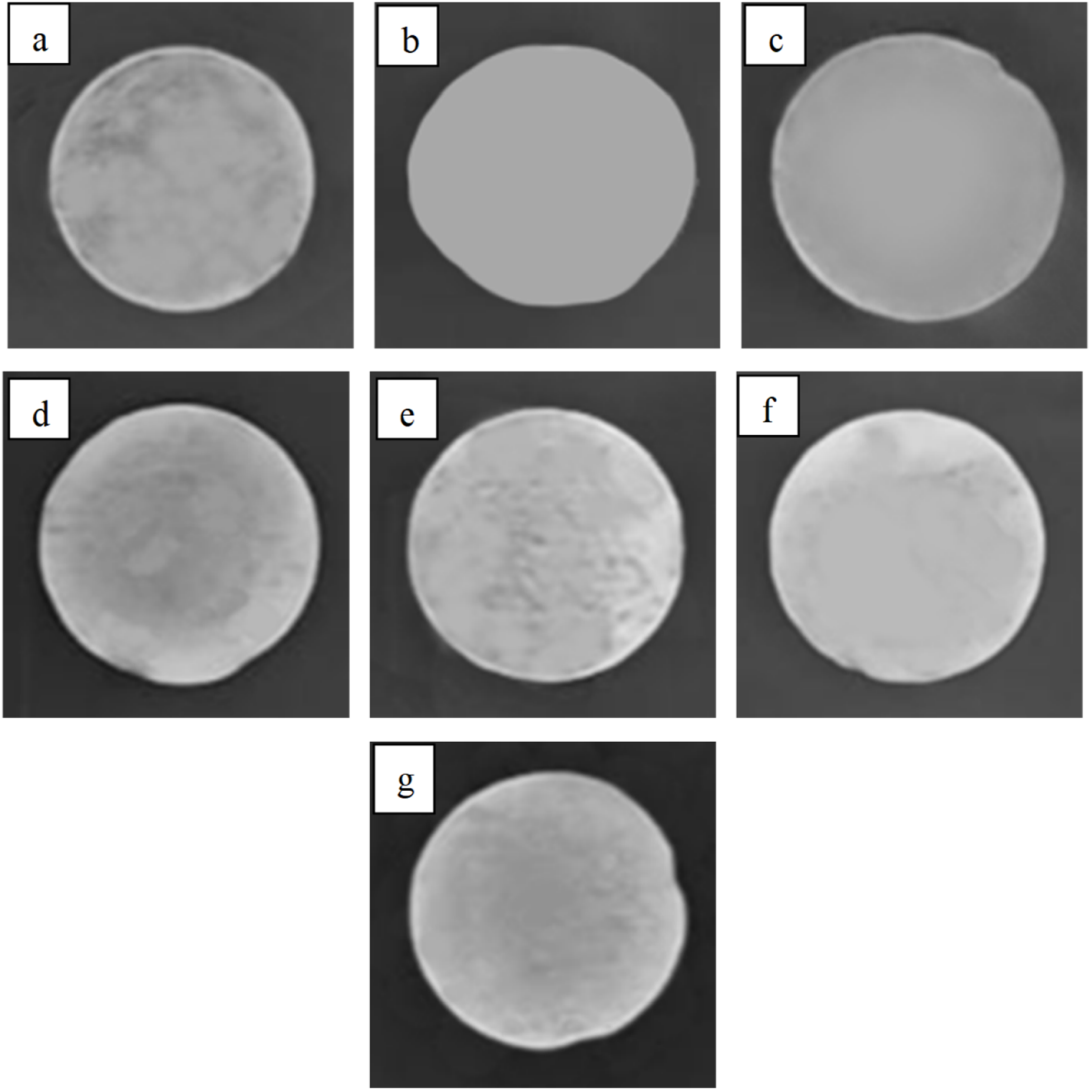
Results of radiopacity testing on ALG/PVA/BaSO_4_ hydrogel variation 70 : 30; (a) Na_2_HPO_4_ 0.5%, (b) BaSO_4_ 1%/Na_2_HPO_4_ 0.3%, (c) BaSO_4_ 1%/Na_2_HPO_4_ 0.4%, (d) BaSO_4_ 1%/Na_2_HPO_4_ 0.5%, (e) BaSO_4_ 1.5%/Na_2_HPO_4_ 0.3%, (f) BaSO_4_ 1.5%/Na_2_HPO_4_ 0.4%, (g) BaSO_4_ 1.5%/Na_2_HPO_4_ 0.5%.

Radiopacity evaluation was conducted to determine the X-ray visibility of the hydrogels, which is critical for biomedical imaging applications. X-ray imaging (Fig. 8) was used to assess image brightness (radiopacity), which increased with BaSO_4_ concentration, consistent with previous studies.^43^ The 1.5 wt% BaSO_4_ sample displayed the highest radiopacity. Furthermore, Hue–Saturation–Brightness (HSB) analysis (Table 4) confirmed this trend, with 76% brightness measured for the 80 : 20 hydrogel ratio.

**Table 4:** The brightness of radiopacity results

Sample	% brightness	
	70 : 30	80 : 20
Na_2_HPO_4_ 0.5%	65	66
BaSO_4_ 1%/Na_2_HPO_4_ 0.3%	66	68
BaSO_4_ 1%/Na_2_HPO_4_ 0.4%	69	70
BaSO_4_ 1%/Na_2_HPO_4_ 0.5%	70	71
BaSO_4_ 1.5%/Na_2_HPO_4_ 0.3%	71	73
BaSO_4_ 1.5%/Na_2_HPO_4_ 0.4%	71	74
BaSO_4_ 1.5%/Na_2_HPO_4_ 0.5%	73	76

### Mechanical characterization

**Fig. 9:**
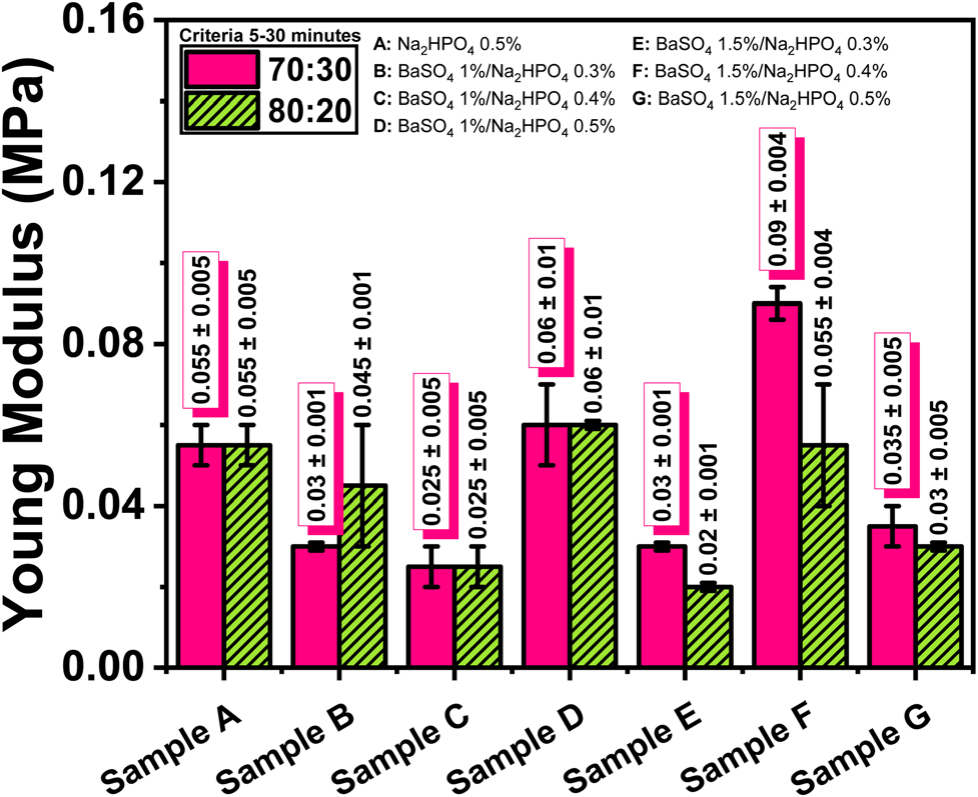
Young modulus values of the ALG/PVA/BaSO_4_ hydrogel composites.

Young's modulus values (Table 5) of all hydrogel composites ranged from 0.020 to 0.090 MPa, which is within the range of native nucleus pulposus (NP) tissue modulus (0.0649 ± 0.044 MPa). Notably, the formulation containing 1.5 wt% BaSO_4_ and 0.4 wt% Na_2_HPO_4_ exhibited the highest modulus (0.090 ± 0.040 MPa), which closely matches the upper bound of the NP modulus range (Fig. 9).

**Table 5:** Young modulus values of the ALG/PVA/BaSO_4_ hydrogel composites

Sample	Young modulus (MPa)	
	0.0649 ± 0.044 MPa (ref. 44)	
	70 : 30	80 : 20
Na_2_HPO_4_ 0.5%	0.055 ± 0.005	0.055 ± 0.005
BaSO_4_ 1%/Na_2_HPO_4_ 0.3%	0.030 ± 0.000	0.045 ± 0.015
BaSO_4_ 1%/Na_2_HPO_4_ 0.4%	0.025 ± 0.005	0.025 ± 0.005
BaSO_4_ 1%/Na_2_HPO_4_ 0.5%	0.060 ± 0.010	0.060 ± 0.000
BaSO_4_ 1.5%/Na_2_HPO_4_ 0.3%	0.030 ± 0.000	0.020 ± 0.000
BaSO_4_ 1.5%/Na_2_HPO_4_ 0.4%	0.090 ± 0.040	0.055 ± 0.015
BaSO_4_ 1.5%/Na_2_HPO_4_ 0.5%	0.035 ± 0.005	0.030 ± 0.000

**Fig. 10:**
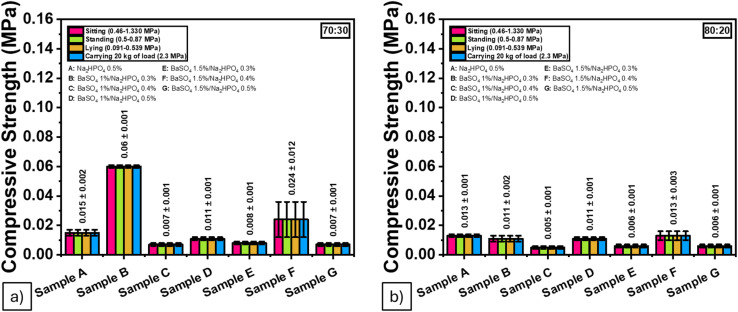
Compressive strength values of the ALG/PVA/BaSO_4_ hydrogel composite from (a) 70 : 30, (b) 80 : 20 ratio.

However, the compressive strength values (Table 6 and Fig. 10) fell short of the mechanical requirements for NP, which range from 0.091 to 1.33 MPa under physiological loading.^45^ Although higher porosity typically weakens mechanical integrity, formulations containing 1.5 wt% BaSO_4_, particularly in the 70 : 30 ratio with 0.4 wt% Na_2_HPO_4_, showed comparatively improved strength. This enhancement may result from better BaSO_4_ particle packing and scaffold densification; however, the values remain below the physiological threshold, indicating the need for further mechanical optimization.

**Table 6:** Compressive strength values of the ALG/PVA/BaSO_4_ hydrogel composite (ap, b, and N refer to alginate/PVA 70 : 30, BaSO_4_, and Na_2_HPO_4_ respectively. The capital letters represent the variation 80 : 20)

Sample	Compressive strength in some conditions for intervertebral disc (MPa)^46^			
	Sitting (0.46–1.330 Mpa)	Standing (0.5–0.87 Mpa)	Lying (0.091–0.539 Mpa)	Carrying 20 kg of load (2.3 Mpa)
apn0.5	0.015 ± 0.002	0.015 ± 0.002	0.015 ± 0.002	0.015 ± 0.002
apb1n0.3	0.006 ± 0.001	0.006 ± 0.001	0.006 ± 0.001	0.006 ± 0.001
apb1n0.4	0.007 ± 0.000	0.007 ± 0.000	0.007 ± 0.000	0.007 ± 0.000
apb1n0.5	0.011 ± 0.001	0.011 ± 0.001	0.011 ± 0.001	0.011 ± 0.001
apb1.5n0.3	0.008 ± 0.000	0.008 ± 0.000	0.008 ± 0.000	0.008 ± 0.000
apb1.5n0.4	0.024 ± 0.012	0.024 ± 0.012	0.024 ± 0.012	0.024 ± 0.012
ap1.5n0.5	0.007 ± 0.001	0.007 ± 0.001	0.007 ± 0.001	0.007 ± 0.001
APN0.5	0.013 ± 0.000	0.013 ± 0.000	0.013 ± 0.000	0.013 ± 0.000
APB1N0.3	0.011 ± 0.002	0.011 ± 0.002	0.011 ± 0.002	0.011 ± 0.002
APB1N0.4	0.005 ± 0.001	0.005 ± 0.001	0.005 ± 0.001	0.005 ± 0.001
APB1N0.5	0.011 ± 0.000	0.011 ± 0.000	0.011 ± 0.000	0.011 ± 0.000
APB1.5N0.3	0.006 ± 0.001	0.006 ± 0.001	0.006 ± 0.001	0.006 ± 0.001
APB1.5N0.4	0.013 ± 0.003	0.013 ± 0.003	0.013 ± 0.003	0.013 ± 0.003
APB1.5N0.5	0.006 ± 0.000	0.006 ± 0.000	0.006 ± 0.000	0.006 ± 0.000

Despite the compressive strengths remaining below NP-relevant levels, the data highlight a complex relationship among porosity, composition, and mechanical behavior. Reinforcement strategies such as nanofibers or hybrid scaffolds are needed for future improvement. These findings suggest a necessary trade-off between achieving optimal porosity for biological integration and sufficient mechanical robustness, which should be carefully balanced in future scaffold designs.

### Biocompatibility assessment

**Fig. 11:**
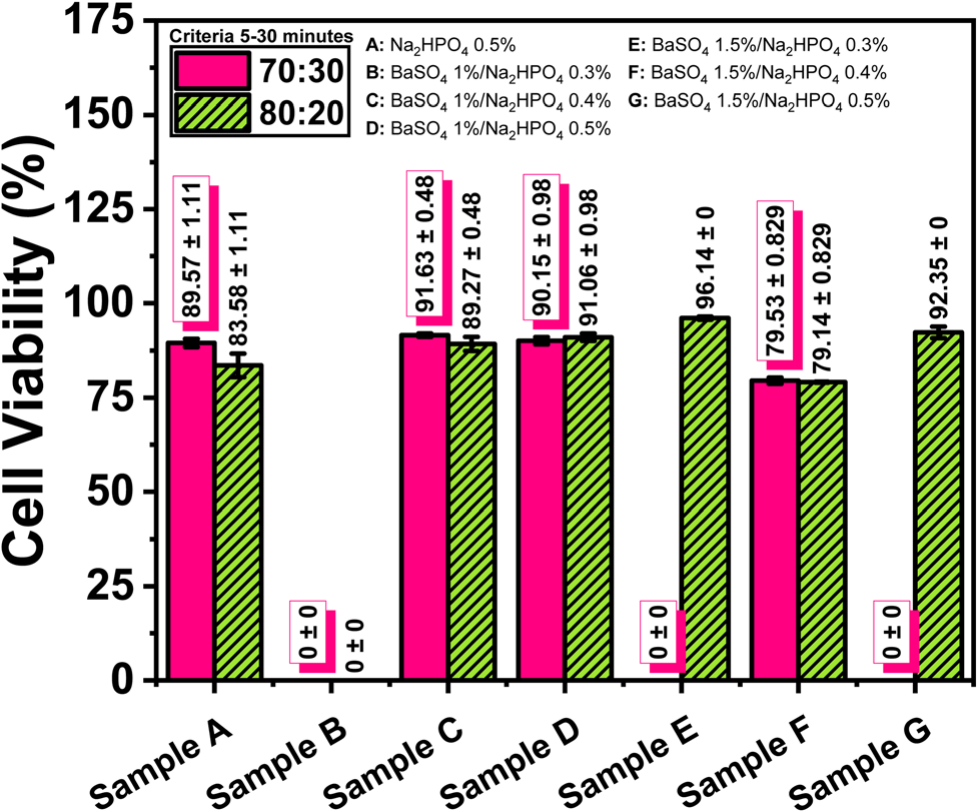
Effect of BaSO_4_ and Na_2_HPO_4_ on cell viability of the ALG/PVA hydrogel composite.

MTT assay results (Table 7 and Fig. 11) demonstrate that most hydrogel formulations achieved high cell viability exceeding 90%, especially those containing 1% BaSO_4_ and 0.5% Na_2_HPO_4_ in both 70 : 30 and 80 : 20 ALG/PVA ratios. In contrast, formulations with 1.5% BaSO_4_ and 0.4% Na_2_HPO_4_ exhibited significantly reduced viability (∼79%), suggesting a possible concentration-dependent cytotoxic response. Several combinations, such as 1% BaSO_4_ with 0.3% Na_2_HPO_4_ and 1.5% BaSO_4_ with 0.5% Na_2_HPO_4_ in the 70 : 30 ratio, resulted in non-detectable or inconsistent viability data (N/A), potentially due to poor dispersion or localized accumulation of BaSO_4_ particles, which has been associated with cytotoxic effects.^47,48^ Despite these anomalies, the overall data support the biocompatibility of the composite, particularly in optimized concentrations that balance radiopacity with cellular health. Among the tested polymer ratios, the 80 : 20 composition generally yielded slightly higher cell viability values, indicating improved compatibility at lower PVA content.

**Table 7:** Effect of BaSO_4_ and Na_2_HPO_4_ on cell viability of the ALG/PVA hydrogel composite

Sample	Cell viability (%)	
	70 : 30	80 : 20
Control positive	100	
Control negative	0	
Na_2_HPO_4_ 0.5%	89.576 ± 1.11	83.576 ± 3.12
BaSO_4_ 1%/Na_2_HPO_4_ 0.3%	N/A	N/A
BaSO_4_ 1%/Na_2_HPO_4_ 0.4%	91.634 ± 0.48	89.269 ± 1.88
BaSO_4_ 1%/Na_2_HPO_4_ 0.5%	90.155 ± 0.98	91.064 ± 0.98
BaSO_4_ 1.5%/Na_2_HPO_4_ 0.3%	N/A	96.139 ± 0.43
BaSO_4_ 1.5%/Na_2_HPO_4_ 0.4%	79.529 ± 0.829	79.137 ± 0.132
BaSO_4_ 1.5%/Na_2_HPO_4_ 0.5%	N/A	92.350 ± 1.526

The two-way ANOVA results (Table 8) indicate that both the sample composition (*p* < 0.001) and the ALG/PVA polymer ratio (*p* = 0.003) exert statistically significant effects on cell viability outcomes. Moreover, a significant interaction was observed between sample composition and polymer ratio (*F* = 5.933, *p* = 0.003), suggesting that the effect of composition on viability is dependent on the specific polymer blend used. The model yielded a high coefficient of determination (adjusted *R*^2^ = 0.906), indicating that over 90% of the variance in cell viability can be explained by these two variables and their interaction. These findings reinforce the importance of fine-tuning both BaSO_4_/Na_2_HPO_4_ concentrations and the polymer blend ratio to maximize biocompatibility in hydrogel scaffold design.

**Table 8:** Two-way analysis of effect of BaSO_4_ and Na_2_HPO_4_ on cell viability of the ALG/PVA hydrogel composite^a^

Source	Type III sum of squares	df	Mean square	F	Sig
Corrected model	1138.889	9	126.543	42.925	0.000
Intercept	295 139.141	1	295 139.141	100 114.007	0.000
Sample_Comp	1078.890	5	215.778	73.194	0.000
Ratio	29.884	1	29.884	10.137	0.003
Sample_Comp*Ratio	52.475	3	17.492	5.933	0.003
Error	88.441	30	2.948		
Total	312 784.601	40			
Corrected total	1227.330	39			

a *R* squared = 0.928 (adjusted *R* squared = 0.906).

### Swelling behavior

**Fig. 12:**
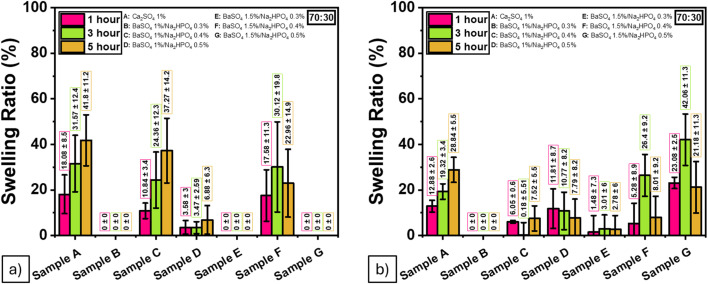
Swelling ratios of the ALG/PVA/BaSO_4_ hydrogel composites at different time points from (a) 70 : 30, (b) 80 : 20 ratio.

The swelling behavior of the hydrogels demonstrated a significant time-dependent increase in water uptake, with swelling ratios ranging from approximately 400% to 800% over a 5-hour immersion period. This substantial increase indicates efficient fluid absorption capacity, a key feature of hydrogels intended for biomedical applications. Maintaining high levels of hydration is critical in nucleus pulposus (NP) regeneration,^49^ as it helps preserve disc height and mechanical function under compressive loads. Therefore, the observed swelling performance reinforces the potential applicability of ALG/PVA/BaSO_4_ composites for intervertebral disc repair. This trend is illustrated in Fig. 12.

### Short-term degradation

**Fig. 13:**
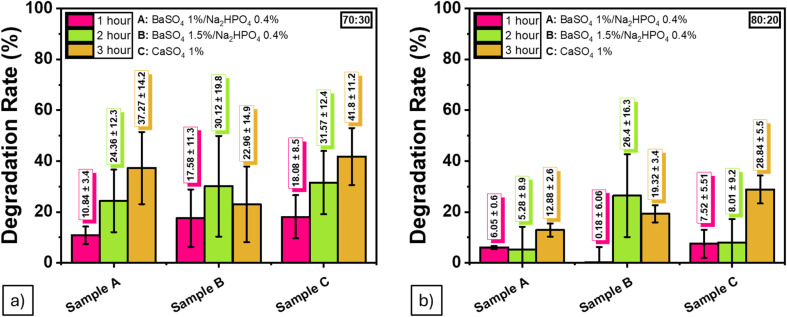
Initial degradation behavior of ALG/PVA/BaSO_4_ hydrogel composites over 5 hours from (a) 70 : 30, (b) 80 : 20 ratio (short-term assessment).

These short-term results are consistent with previous studies reporting rapid water uptake in superabsorbent hydrogels.^50,51^ While this experiment did not assess long-term biodegradation, the observed early-stage degradation behavior offers meaningful insights into hydrogel stability and assists in identifying suitable formulations for future durability studies (Table 9 and Fig. 13).

**Table 9:** Initial degradation behavior of ALG/PVA/BaSO_4_ hydrogel composites over 5 hours (short-term assessment)

Duration	Degradation rate (%)		
	BaSO_4_ 1%/Na_2_HPO_4_ 0.4%	BaSO_4_, 1.5%; Na_2_HPO_4_, 0.4%	CaSO_4_ 1%
**70 : 30**			
1 hour	10.84 ± 3.4	17.58 ± 11.3	18.08 ± 8.5
3 hour	24.36 ± 12.3	30.12 ± 19.8	31.57 ± 12.4
5 hour	37.27 ± 14.2	22.96 ± 14.9	41.80 ± 11.2

**80 : 20**			
1 hour	6.05 ± 0.6	5.28 ± 8.9	12.88 ± 2.6
3 hour	−0.18 ± 6.06	26.40 ± 16.3	19.32 ± 3.4
5 hour	7.52 ± 5.51	8.01 ± 9.2	28.84 ± 5.5

The overarching objective of this study was to develop an injectable, biocompatible, and radiopaque hydrogel composite suitable for nucleus pulposus (NP) regeneration. Each of the experimental findings—gelation time, structural analysis, mechanical performance, swelling behavior, degradation rate, and cytocompatibility—collectively supports the feasibility of this formulation for clinical application. Optimized concentrations of Na_2_HPO_4_ and BaSO_4_ yielded short gelation times (8.5–15.5 min), which fall within the recommended range for *in situ* injectability during minimally invasive spinal procedures (5–30 minutes). FTIR results demonstrated that the crosslinking process is dominated by physical interactions, as evidenced by unaltered characteristic peaks of alginate and PVA, thereby preserving the chemical stability of the base polymers and confirming the bioinert nature of BaSO_4_.

Morphological observations revealed that hydrogels containing 1.5 wt% BaSO_4_ and 0.4 wt% Na_2_HPO_4_ exhibited the highest porosity and pore sizes, which are considered favorable for biomedical scaffolds. Such microstructures facilitate cell infiltration and nutrient diffusion, essential for tissue integration and long-term scaffold performance.^41^ SEM and EDS analysis also revealed BaSO_4_ agglomeration in some samples, suggesting the need for improved interfacial compatibility between filler particles and the polymer matrix to ensure consistent dispersion and mechanical uniformity.

Radiopacity increased in direct proportion to BaSO_4_ content, enabling clear X-ray visualization of the hydrogel implants—a critical requirement for monitoring implant placement and integrity during spinal procedures. Mechanical analysis showed that while the elastic modulus of several formulations (ranging from 0.025 to 0.090 MPa) fell within the acceptable range for native NP tissue (0.0649 ± 0.044 MPa), the compressive strengths remained well below physiological benchmarks (0.091–1.33 MPa). This mismatch highlights the need for future reinforcement strategies—such as incorporating reinforcing nanofillers or developing dual-network systems—to improve the hydrogel's load-bearing capacity.

Biocompatibility assessments revealed that formulations containing 1 wt% BaSO_4_ and either 0.4% or 0.5% Na_2_HPO_4_ maintained high cell viability above 90%, indicating favorable cytocompatibility. However, formulations with 1.5 wt% BaSO_4_—especially at 0.4% Na_2_HPO_4_—exhibited reduced viability (∼79%), suggesting potential cytotoxic effects at higher BaSO_4_ concentrations. Swelling and degradation analyses confirmed the hydrogel's strong water absorption and gradual mass loss under simulated physiological conditions, both of which are essential for maintaining hydration and replicating the functional environment of the native nucleus pulposus.

Overall, the results highlight a complex but consistent relationship between the hydrogel's chemical composition and its functional performance. Tuning the concentrations of BaSO_4_ and Na_2_HPO_4_ not only improved gelation time and radiopacity but also influenced key parameters such as mechanical strength, porosity, and cytocompatibility. This interdependent behavior among formulation variables and performance metrics supports the hydrogel's potential as an injectable, biocompatible scaffold with radiopaque visibility for nucleus pulposus regeneration, while also emphasizing the need for further mechanical optimization.

## Conclusion

This study aimed to evaluate the effects of varying concentrations of Na_2_HPO_4_ (0.3%, 0.4%, and 0.5%) and BaSO_4_ (1% and 1.5%) on the physical, mechanical, and biological performance of ALG/PVA composite hydrogels as injectable scaffolds for nucleus pulposus (NP) regeneration. The optimal formulation—1.5 wt% BaSO_4_ and 0.4 wt% Na_2_HPO_4_—demonstrated a gelation time of 12.5 ± 0.5 minutes, an elastic modulus of 0.055 ± 0.015 MPa, radiopacity of 71–74%, porosity above 80%, and cell viability exceeding 90%, thereby satisfying key criteria for NP applications. All hydrogel variations exhibited gelation times within the clinically acceptable range (5–30 minutes). The incorporation of BaSO_4_ enhanced radiopacity in proportion to its concentration, while Na_2_HPO_4_ effectively modulated gelation kinetics.

Although elastic modulus values improved, the compressive strength (maximum 0.024 ± 0.012 MPa) remained below the physiological threshold (0.091–1.33 MPa), indicating a need for further mechanical enhancement. This study offers a promising foundation for the development of multifunctional hydrogels; however, future work should focus on improving mechanical load-bearing capacity through composite reinforcement and validating long-term *in vivo* performance.

*In vitro* biocompatibility tests using the MTT assay on osteoblast-like 7F2 cells showed that formulations with 0.4% and 0.5% Na_2_HPO_4_ achieved high cell viability (>90%), confirming favorable cytocompatibility. The formulation with 1% BaSO_4_ and 0.5% Na_2_HPO_4_ exhibited the highest viability (91.064 ± 0.98%). These results support the hydrogel's suitability for biomedical use, particularly in tissue engineering and regenerative medicine.

The swelling behavior showed a significant time-dependent increase in water absorption, supporting the hydrogel's ability to maintain hydration and structural integrity in the disc space. Additionally, degradation studies revealed that CaSO_4_ decomposed faster than BaSO_4_, emphasizing the importance of selecting appropriate radiopaque and retarding agents to tailor *in vivo* performance.

The dual incorporation of Na_2_HPO_4_ and BaSO_4_ in a single system represents a novel approach that simultaneously addresses gelation kinetics and radiopacity requirements for injectable NP scaffolds.

In summary, while the hydrogel system shows strong potential in terms of biocompatibility, gelation behavior, and imaging visibility, future research must address mechanical reinforcement to fully meet clinical load-bearing demands.

## Conflicts of interest

There are no conflicts to declare.

## Data Availability

Due to the confidential nature of the data, they are not publicly available. Data may be available from the corresponding author on reasonable request and subject to confidentiality agreements.
